# Workload and quality of nursing care: the mediating role of implicit rationing of nursing care, job satisfaction and emotional exhaustion by using structural equations modeling approach

**DOI:** 10.1186/s12912-022-01055-1

**Published:** 2022-10-08

**Authors:** Fatemeh Maghsoud, Mahboubeh Rezaei, Fatemeh Sadat Asgarian, Maryam Rassouli

**Affiliations:** 1 Medical Surgical Nursing, School of Nursing and Midwifery, Kashan University of Medical Sciences, Kashan, Iran; 2 Trauma Nursing Research Center, School of Nursing and Midwifery, Kashan University of Medical Sciences, 5th of Qotb –e Ravandi Blvd, P.O.Box: 8715981151, Kashan, Iran; 3 Social Determinants of Health (SDH) Research Center, Kashan University of Medical Sciences, Kashan, Iran; 4 Department of Pediatric and Reproductive Health, School of Nursing and Midwifery, Shahid Beheshti University of Medical Sciences, Tehran, Iran

**Keywords:** Workload, Health care rationing, Job satisfaction, Quality of nursing care, Structural equation modeling

## Abstract

**Background:**

Nursing workload and its effects on the quality of nursing care is a major concern for nurse managers. Factors which mediate the relationship between workload and the quality of nursing care have not been extensively studied. This study aimed to investigate the mediating role of implicit rationing of nursing care, job satisfaction and emotional exhaustion in the relationship between workload and quality of nursing care.

**Methods:**

In this cross-sectional study, 311 nurses from four different hospitals in center of Iran were selected by convenience sampling method. Six self-reported questionnaires were completed by the nurses. The data were analyzed by SPSS version 16. Structural equation modeling was used to determine the relationships between the components using Stata 14 software.

**Results:**

Except direct and mutual relationship between workload and quality of nursing care (*P* ≥ 0.05), the relationship between other variables was statistically significant (*P* < 0.05). The hypothesized model fitted the empirical data and confirmed the mediating role of implicit rationing of nursing care, job satisfaction and emotional exhaustion in the relationship between workload and the quality of nursing care (TLI, CFI > 0.9 and RMSEA < 0.08 and χ^2^/df < 3).

**Conclusion:**

Workload affects the quality of the provided nursing care by affecting implicit rationing of nursing care, job satisfaction and emotional exhaustion. Nurse managers need to acknowledge the importance of quality of nursing care and its related factors. Regular supervision of these factors and provision of best related strategies, will ultimately lead to improve the quality of nursing care.

## Background

Care is the core of the nursing profession and the main factor which distinguishes nursing from other health-related professions [1, 2]. High-quality nursing care means the provision of easy and accessible care by competent qualified nurses [3]. Nowadays, the maintenance and improvement of the quality of nursing care is the most important challenge for nursing care systems around the world [4]. The first step in improving the quality of nursing care is to evaluate and analyze the quality of provided care and examine the factors affecting on it [5].

Various variables can affect the quality of nursing care [6–8]; one of which is workload. Zuniga et al. (2015) indicated in Switzerland that increased workload and, subsequently, increased stress could reduce the quality of nursing care [9]. However, there are contradictory findings in this regard. It was shown in another study that there was a high level of nursing care quality despite the high workload and inadequate human resources and equipment [6]. In another study, the workload was measured by total direct nursing hours. The results showed a significant correlation between total direct nursing hours and some indicators of nursing care quality such as incidence of patient restraint, and mortality rate. Nevertheless, there was no significant correlation with other indicators of nursing care quality like incidence density of pressure sores, the incidence of falls, the incidence of tube self-extraction, and incidence density of infection [10].

In addition to the correlation between workload and the quality of nursing care, a number of other factors can also be involved in this relationship. For example, workload can lead to implicit rationing of nursing care, thereby can affect the quality of care. In a study conducted in Lebanon, the level of perceived workload in all shifts had a positive relationship with the level of rationing of nursing care [11]. Because of many reasons such as high workload, nurses may find themselves in situations where they are forced to omit the necessary cares, do them briefly or with delay [11, 12]. Nurses are unable to provide comprehensive care in accordance with professional standards, and it can affect the quality of nursing care [13]. A study conducted in China showed that the nurses who had a higher score in rationing of nursing care, had a lower score of the quality of nursing care [14]. Moreover, while increased rationing in rehabilitation, care, supervision and social care in nursing homes, decreases the quality of nursing care, increased rationing in the field of documentation increases the quality of nursing care [9].

Job satisfaction seems to be another factor mediating the relationship between workload and the quality of nursing care. Inegbedion et al. (2020) indicated that increased workload could be associated with decreased job satisfaction among nurses [15]. Workload as a strong stressor can negatively affect the job satisfaction of nurses [16]. Job satisfaction is a multidimensional emotional concept which reflects the interaction between nurses' expectations and values, their environment and personal characteristics [17]. Perception of the significance of nurses' job satisfaction and its improvement is essential in providing high-quality care with optimal clinical outcomes. In the study of Aron et al. (2015), 87.6% of nurses believed that the quality of care provided by nurses was affected by their job satisfaction [18]. According to another study, job satisfaction was a significant predictor of the quality of nursing care [19].

Workload may also affect the quality of nursing care by causing emotional exhaustion in nurses. The results of a study revealed that 55.4% of Canadian nurses suffered from emotional exhaustion. The high workload in this study was a predictor of emotional exhaustion and there was a positive and significant correlation between workload and emotional exhaustion [20]. Additionally, the findings of Nantsupawat et al. (2016) were indicative of the effect of emotional exhaustion on the quality of nursing care. While increased emotional exhaustion of nurses in their study increased the incidence of medication errors and infections, it decreased the quality of nursing care [21]. Findings of another study showed that among the components of job burnout, emotional exhaustion had the strongest relationship with the quality of nursing care [22].

Previous studies have mainly investigated the relationship of one or two variables with the quality of nursing care and the simultaneous effect of several mediating variables on the quality of nursing care has not been examined [23–25]. Many of these studies have not used a comprehensive questionnaire to assess all aspects of the quality of nursing care or have been conducted in other settings except hospital units [6, 9, 26, 27]. Assessing the quality of nursing care with an incomplete questionnaire or with only one question does not cover all dimensions of quality of nursing care such as the care-related activities, nursing care environment, nursing process, and strategies that empower patients and will provide incomplete findings [28, 29].

Accordingly, to improve the quality of nursing care, we need to determine these variables and their mediating roles, in order to better control them through applying effective interventions. Using Structural Equation Modeling (SEM) is one powerful tool for mediation analysis [30, 31], this study was conducted to investigate the mediating role of implicit rationing of nursing care, job satisfaction, and emotional exhaustion in the relationship between workload and the quality of nursing care in Iran.

### Hypotheses

**Fig. 1:**
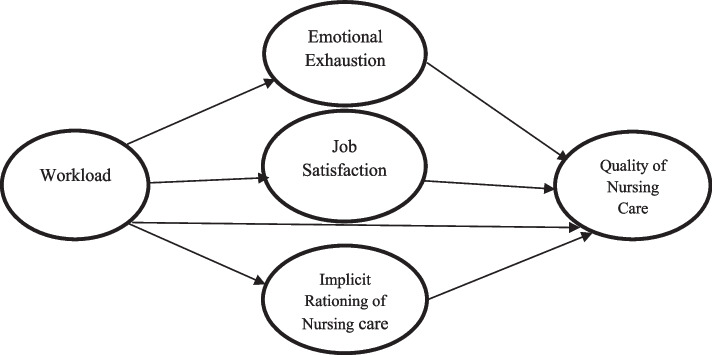
Hypothesized model

The theoretical model in this study was developed by reviewing the related literature (Fig. 1) to test three hypotheses:

H1: Implicit rationing of nursing care plays a mediating role in the relationship between workload and the quality of nursing care.

H2: Job satisfaction plays a mediating role in the relationship between workload and the quality of nursing care.

H3: Emotional exhaustion plays a mediating role in the relationship between workload and the quality of nursing care.

## Methods

### Study design and participants

This cross-sectional study was conducted from October to December 2020 in inpatient units of four selected hospitals in central Iran, Kashan city. According to the guidelines of structural equation modeling, the study required at least 300 participants [32]. As such, 311 employed nurses participated in the study by using the convenience sampling method. Inclusion criteria were as follows: willingness to participate in the study, having at least six months of work experience, having experience of direct clinical care of patients, and having at least a bachelor's degree in nursing. Exclusion criteria were failure to complete the questionnaire and decline to answer the questionnaires in the process of the study.

### Instrumentation

Six tools were used to collect data and analyze the variables of this study:

1. Nurse's demographic information questionnaire which contains questions about age, gender, marital status, current workplace unit, employment status, nursing work experience, duration of working in the current unit, having overtime, average salary per month, being a nurse as a second job, having a second job beside nursing and the level of interest in the nursing.
2. The NASA Task Load Index (NASA-TLX) includes six areas of mental demand, physical demand, temporal demand, performance, effort and, frustration. The final score is calculated to be between zero and 100, where scores higher than 50 are indicative of a high overall subjective workload [33]. Using Cronbach's alpha coefficient, the reliability of this questionnaire has been reported to be above 0.8 in previous studies [34, 35].
3. Basel Extend of Rationing of Nursing Care (BERNCA) questionnaire which has 20 items based on a 4-point Likert scale. In this questionnaire, nurses assess themselves how many times in the past month they have not been able to perform the listed care activities and have been forced to ration them. The total mean score of rationing is 0–3, and the higher the score, the more will be the care that has been rationed. Cronbach's alpha coefficient was calculated to be 0.93 [36]. The reliability coefficient was calculated at 0.91 in the present study.
4. The Minnesota Satisfaction Questionnaire (MSQ) which was designed by Weiss et al. (1967) and has two long and short versions [37]. In this study, the short version of the questionnaire was used. This 18-item questionnaire is based on a 5-point Likert scale and higher scores are indicative of better job satisfaction. The reliability and validity of this questionnaire was determined in Iran [38]. Using Cronbach's alpha, the reliability of this questionnaire was calculated at 0.77 in the present study.
5. The emotional exhaustion subscale of the Maslach Burnout Inventory (MBI), includes nine items and is based on a 7-point Likert scale. Higher scores indicate higher emotional exhaustion [39]. The validity and reliability of this scale were examined in Iran and Cronbach's alpha coefficient was reported to be 0.88 [40]. Cronbach's alpha coefficient was calculated to be 0.90 in the study of Maslach et al. (1996) and 0.89 in the present study.
6. The Good Nursing Care Scale (GNCS) is a comprehensive questionnaire that examines all aspects of the quality of nursing care. It has two parallel versions for the nurse and the patient and the nurse's version was used in the present study. This questionnaire has 40 items and seven dimensions include nurses’ characteristics in providing care ( *such as type of interaction with the patient, and accuracy*), care-related activities (*such as patient education, and emotional support*), care preconditions (*such as nurse’s knowledge, skill, and experience*), nursing care environment (*such as infection control, maintain patient safety, and patient privacy protection*), nursing process (*conditions related to patient’s admission, treatment and, discharge*), patient empowerment strategies in coping with the disease (*such as paying attention to the patient’s level of knowledge, answering the questions*), and collaboration with the patient's family and relatives (*such as providing sufficient information to the family, and family participation in treatment process*). The scale is based on a 5-point Likert scale and the higher the obtained score, the more will be the quality of provided care [28, 29]. This scale has been psychometrically evaluated and used in different countries and Cronbach's alpha coefficient for the scale has been in the range of 0.80 to 0.94 in various studies [6, 41–43]. Cronbach's alpha coefficient was calculated to be 0.93 in the present study.

### Data collection

All six questionnaires were filled out by the participants based on their work performance in the past month. After obtaining the informed written and oral consent of the eligible nurses, they were explained how to complete the questionnaires. It order to prevent the nurses’ fatigue, the questionnaires were prepared in both online and paper format. The researcher asked each of the participants if they wanted to fill out the questionnaires online or on paper format. If the participants chose the paper format, the questionnaires were delivered to them and were collected at the appointed time. All of the questionnaires have been assessed immediately after the response of the participants and any missing data have been filled by them. But if the participants selected the online version, the link to the questionnaires was sent to their cellphone. This link was designed in such a way that a person could answer only once through the link of the questionnaire and until all the questions were answered, the questionnaire was not sent. Accordingly, there were no missing data. All data collection process was done by a researcher (first author).

### Data analysis

Data were analyzed using SPSS version 16 and Stata version 14. Categorical data were described by frequencies and percentages, and quantitative continuous data by mean and standard deviation (SD). The correlation between the variables was determined by the Pearson correlation coefficient test. Structural equation modeling was used to capture the structure of relationships among a web of latent and observed components. To understand the relationships between the variables, according to the theoretical model of the study, all variables were analyzed using Stata software and the structural model was developed. In this study, three types of absolute, comparative and, parsimony fit indices were examined. The Root Mean Square Error of Approximation (RMSEA), the Comparative Fit Index (CFI) Tucker-Lewis Index (TLI), and the ratio of chi-square to the degrees of freedom (χ2 / df) were considered for the good fit of the model. A model is considered to have good fit if the (χ2 / df) value is lower than 3, CFI and TLI are 0.90 or greater, and the RMSEA value is less than 0.08 [44, 45]

## Results

### Participants’ characteristics

All of 311 distributed questionnaires were completed and analyzed. The mean age of the nurses participating in the study was 32.68 ± 6.73 years. The majority of the participants were female (86.5%) and married (76.2%). The complete demographic information of the participants can be seen in Table 1.

**Table 1:** The characteristics of the participants (*n* = 311)

Characteristics		No. (%)
Gender	Female	269 (86.5)
	Male	42 (13.5)
Marital status	Single	70 (22.5)
	Married	237 (76.2)
	Divorced	3 (1)
	Widow	1 (0.3)
Current workplace unit	Internal	152 (48.9)
	Surgery	32 (10.3)
	Pediatrics	11 (3.5)
	Neonatal	15 (4.8)
	Obstetrics and gynecology	13 (4.2)
	ICU/CCU	88 (28.3)
Employment status	Permanent	189 (60.7)
	contractual	122 (39.3)
Overtime	Yes	290 (93.2)
	No	21 (6.8)
Being a nurse as a second job	Yes	2 (0.6)
	No	309 (99.4)
Having a second job beside nursing	Yes	17 (5.5)
	No	294 (94.5)
Interest to the nursing profession	Not at all	3 (1)
	Low	8 (2.6)
	Moderate	114 (36.6)
	High	130 (41.8)
	Very high	56 (18)
Age (years) (Mean ± SD)	32.68 ± 6.73	
Duration of working in the current unit (months) (Mean ± SD)	54.35 ± 54.61	
Experience of working as a nurse (months) (Mean ± SD)	105.18 ± 73.50	
Average amount of salary per month (Million Tomans) ^a^ (Mean ± SD)	4.54 ± 0.98	

^a^Iran currency

### Bivariate analysis

According to the scoring of the questionnaires, the workload of the nurses was at a high level, implicit rationing of nursing care happened rarely, the job satisfaction of nurses was at a moderate level and emotional exhaustion was at a low level. Moreover, the quality of the provided nursing care was at a good level (Table 2).

**Table 2:** Mean and standard deviation, minimum and maximum scores of the research variables

Variable	Mean	SD	Min	Max
Workload	65.37	13.54	13.66	96
Implicit rationing of nursing care	1.11	0.59	0	3
Job satisfaction	56.44	10.91	21	90
Emotional exhaustion	20.66	12.42	0	54
Quality of nursing care	3.16	0.45	1.75	3.98

According to the result of Pearson correlation coefficient, there was a statistically significant correlation between the various variables of the study (except workload and quality of nursing care) (Table 3).

**Table 3:** Correlation of the research variables

	Quality of nursing care	Implicit rationing of nursing care	Emotional exhaustion	Job satisfaction	Workload
Quality of nursing care	1				
Implicit rationing of nursing care	-0.354^**^	1			
Emotional exhaustion	-0.196^**^	0.495^**^	1		
Job satisfaction	0.228^**^	-0.359^**^	-0.567^**^	1	
Workload	0.059	0.247^**^	0.335^**^	-0.300^**^	1

^*^Significance *P* < 0.05

^**^Significance *P* < 0.01

### Structural equation model

Based on the results of this study, the direct effect of workload on implicit rationing of nursing care, job satisfaction and emotional exhaustion was statistically significant (*p* < 0.05). Moreover, implicit rationing of nursing care, job satisfaction, and emotional exhaustion had an indirect statistically significant effect on the relationship between workload and quality of nursing care (*p* < 0.05) (Table 4).

**Table 4:** Direct and indirect effect of variables

Variable	Direct effect on workload				Indirect effects (mediating role) between workload and quality of nursing care			
	b	SE	Z	P-value	b	SE	Z	P-value
Implicit rationing of nursing care	0.056	0.013	4.21	0.0001	0.027	0.048	5.72	0.001
Job satisfaction	-0.241	0.043	5.55	0.0001	0.031	0.011	0.07	0.001
Emotional exhaustion	0.307	0.049	6.27	0.0001	0.30	0.008	3.69	0.001

*B* Regression Coefficient, *SE* Standard Error

The obtained good fit indices confirmed the mediating role of implicit rationing of nursing care (TLI = 0.94; CFI = 0.95; RMSEA = 0.05), job satisfaction (TLI = 1; CFI = 1; RMSEA = 0.01) and emotional exhaustion (TLI = 0.96; CFI = 0.95; RMSEA = 0.01) in the relationship between workload and the quality of nursing care.

**Fig. 2:**
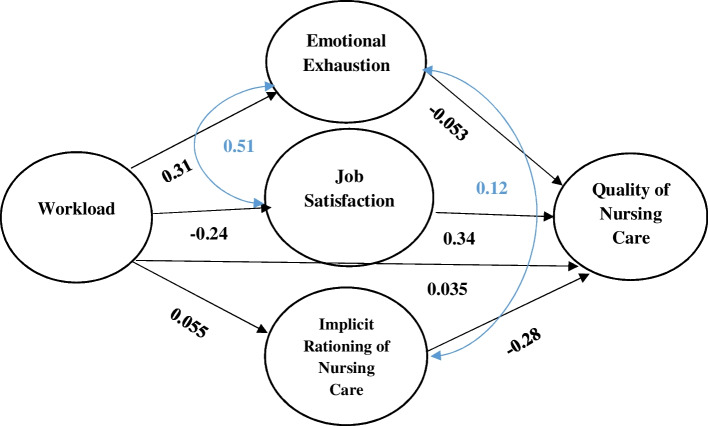
The final model

As shown in Fig. 2, the model fit the data well and was consistent with the hypothesized model. By putting together the three variables of implicit rationing of nursing care, job satisfaction, and emotional exhaustion as mediators in the model, a good fit was obtained (TLI = 0.95; χ2/df = 2.3; CFI = 0.96; and RMSEA = 0.05).

## Discussion

The results of this study supported the proposed hypothesized model. The findings shown in this structural equation model provided strong support for the study hypotheses.

Based on the findings, implicit rationing of nursing care played a mediating role in the relationship between workload and the quality of nursing care. Therefore, the H1 hypothesis was supported. When the nurses’ workload is high and they are responsible for caring for a large number of patients, they are inevitably forced to ration some important interventions which, in turn, can reduce the quality of nursing care [12, 13]. An earlier study showed that implicit rationing of nursing care functions as a mediator between predictive variables such as workload and patient-related outcomes such as medication error and patients’ falling. These adverse events can reduce the quality of nursing care [20]. In other words, workload has an indirect effect on patient-related outcomes through care rationing and affecting the ability of nurses in completing their main tasks. In another study, nurse-to-patient ratio, as an important indicator in the workload of nurses, affected the quality of care and the incidence of adverse events through rationing of care. In other words, poor nurse staffing levels leads to the rationing of nursing care and, thereby, hinders the provision of high quality care [14]. Some other studies also referred to the mediating role of rationing of nursing care in the relationship between workload and patient safety [46] as well as in the relationship between workload and patients’ falling [47]. Accordingly, implicit rationing of nursing care seems to play a key role in the relationship between workload and the quality of nursing care.

In the present study, nurses’ job satisfaction was the second variable that mediated the relationship between workload and quality of nursing care. Hence, the H2 hypothesis was supported. When the workload is increased, nurses cannot meet some of the needs of patients despite the effort they make. So, nurses do not have a positive attitude toward their performances, leading to less job satisfaction [23, 48]. In these circumstances, nurses do not have the necessary peace of mind and precision in the workplace which may negatively affect their efficiency and performance, decreasing the quality of the provided care [49]. Job satisfaction is an important variable that mediates the relationship between workload and other variables such as intention to leave the job and position [50, 51]. Therefore, this is affecting the quality of nursing care indirectly. However, more research is required to investigate the mediating role of job satisfaction.

Emotional exhaustion was another mediating variable in the relationship between workload and quality of nursing care in this study. Therefore, the H3 hypothesis was supported. Emotional exhaustion is considered to be the most important component of job burnout and nurses who experience high levels of emotional exhaustion will suffer from job burnout and have a lower ability and tendency to provide high-quality care [39, 52]. According to Van Bogaert et al. (2009), emotional exhaustion plays a mediating role in the relationship between nurses’ workplace conditions and the quality of nursing care [53]. Liu et al. (2018) also indicated that emotional exhaustion mediates the relationship between workload and patient safety. When nurses are constantly exposed to stressful work environments, their reactions become more chronic and serious, and they need more time to recover [46]. Additionally, because of high workload and regular attendance at the hospital, nurses do not have much opportunity to rest and regain their energy and, thus, will experience a perpetual emotional exhaustion [54].

It is noticed from this study that there was no significant correlation between workload and quality of nursing care. This finding is interesting and in line with an earlier study in which despite the high levels of workload and insufficiency of human resources and equipment, the quality of nursing care was at a high level [6]. Considering that both the nurse’s workload and the quality of nursing care have been investigated from the nurse's point of view, more reliable results have been obtained in this study. It should be noted that the final model in this study is a full mediation model, as the three variables (rationing of nursing care, job satisfaction, and emotional exhaustion) fully mediate the effect of workload on the quality of nursing care. So, after controlling for this mediation effect, there is no direct effect of workload on quality of nursing care [55].

Also, it seems that experience of high levels of workload for a long period of time and fall into the habit of these conditions lead to nurses can manage difficult situations. According to this finding, it is suggested that temporary or permanent high nursing workload should be taken into consideration in the next researches. Also, social desirability bias which is the tendency to respond in a pleasing way, in answering the questions related to quality of nursing care may also have been influential.

### Study limitations

This study has several limitations: a) the present study was confined to frontline nurses in four selected governmental hospitals in a small city in the country; so, generalization of the findings may be limited; b) given the limited number of available participants, the convenience sampling method was used to provide the minimum sample size. It is suggested that future studies be conducted in different cities of the country and private and public hospitals with more participants, to be able to compare the findings; c) the use of self-report questionnaires and nurses’ perceptions to obtain data on the study variables may be a potential limitation because of social desirability bias [56, 57]. It is recommended that future studies use more precise data collection strategies with observation or retrospective methodology; d) because the questionnaires filled out by the participants based on their work performance in the past month, recall bias may be another limitation of this study; e) although SEM approach was used in this study, causation cannot be established with the cross-sectional study design.

### Implication for clinical practice

Nurse managers have a prominent position related to issues such as nursing workload, rationing of nursing care, job satisfaction and emotional exhaustion. Providing any intervention in these fields, will ultimately effect on the quality of nursing care. Nursing workload, which was at high level, is a major concern in this study. Some strategies such as staffing and resource adequacy assessment and hiring more nurses to increase staffing levels can be useful [58]. Moderate levels of job satisfaction and emotional exhaustion were another important finding of this study. Administering flexible work schedules, increasing monthly salary, suggesting some mental health resources, and modification of work environment may be improving nurses’ job satisfaction and decrease their emotional exhaustion. Regular supervision of clinical nursing care activities and provision of continuous feedback are important to ensure that essential nursing care tasks are provided and prevent any compromise of nursing care [59]**.**

## Conclusion

Despite the limitations, this study highlighted that the three variables of implicit rationing of nursing care, job satisfaction and emotional exhaustion played a mediating role in the relationship between workload and quality of nursing care. In other words, the effect of workload was applied to the quality of nursing care through these three variables. Therefore, the assumed theoretical model was approved and provided a theoretical basis for quality of nursing care. Nurse managers should pay special attention to the three mediating variables and monitor them periodically and try to solve problems in these areas. Nurse managers should be aware that support of nurses means the support of high-quality care of the patients and overlooking the issues and problems of nurses will have negative effect on the patients.

## Data Availability

The datasets generated during and/or analyzed during the current study are available from the corresponding author on reasonable request.

## Abbreviations

BERNCA: Basel Extend of Rationing of Nursing Care
CFI: Comparative Fit Index
GNCS: Good Nursing Care Scale
MBI: Maslach Burnout Inventory
MSQ: Minnesota Satisfaction Questionnaire
NASA-TLX: NASA Task Load Index
RMSEA: Root Mean Square Error of Approximation
SD: Standard Deviations
TLI: Tucker-Lewis Index
